# Psychological Aspects of Androgen Insensitivity Syndrome: Two Cases Illustrating Therapeutical Challenges

**DOI:** 10.1155/2017/8313162

**Published:** 2017-03-12

**Authors:** Filippa Pritsini, Georgios A. Kanakis, Ioannis Kyrgios, Eleni P. Kotanidou, Eleni Litou, Konstantina Mouzaki, Aggeliki Kleisarchaki, Dimitrios G. Goulis, Assimina Galli-Tsinopoulou

**Affiliations:** ^1^Unit of Pediatric Endocrinology, 4th Department of Pediatrics, Medical School, Faculty of Health Sciences, Aristotle University of Thessaloniki, Thessaloniki, Greece; ^2^Unit of Reproductive Endocrinology, 1st Department of Obstetrics and Gynecology, Medical School, Faculty of Health Sciences, Aristotle University of Thessaloniki, Thessaloniki, Greece

## Abstract

Androgen Insensitivity Syndrome (AIS) and its heterogeneous phenotypes comprise the pieces of a challenging clinical problem. The lack of standardized guidelines results in controversies regarding the proper diagnostic and therapeutic approach, including the time and type of intervention. Due to its variable phenotype, AIS is not diagnosed at the proper age that would allow optimal psychological and medical support to the patient. Therapeutic approaches are not established, mainly due to the rarity of the disease. In addition, various social and ethical consequences may emerge. The aim of this double case report is to outline the difficulties that may rise during diagnostic, therapeutic, and psychological approach of AIS, especially concerning the handling of the relatives' reaction.

## 1. Introduction

Androgen Insensitivity Syndrome (AIS) is a Disorder of Sexual Differentiation (DSD), characterized by variable target tissue resistance to androgens. It is caused by mutations in the Androgen Receptor (AR) gene, which is located on the long arm of the X chromosome, or by defects of AR signaling, including interacting proteins and coregulatory factors [1–4]. In the majority of the cases, the pattern of inheritance is X-linked recessive; however, occasional de novo mutations have been described. More than 500 mutations have been identified in the AR gene, described in detail in the AR gene mutation database, which is available online at http://androgendb.mcgill.ca/ [accessed 6 September 2016] [5].

As a consequence of AR dysfunction, androgens cannot exert their effects, even since the intrauterine life, resulting in malformations of internal and external genital structures as well as of secondary sexual development and maturation [2]. Depending on the functionality of the AR, the clinical phenotype may vary, ranging from complete feminization to incomplete types with minor degrees of undervirilization or infertility. Accordingly, AIS is classified into three main categories: Complete (CAIS), Partial (PAIS), and Mild (MAIS) (Table 1). Individuals affected by CAIS are born unambiguously female, without any apparent abnormalities. They are usually diagnosed during puberty due to primary amenorrhea. Sexual hair is typically lacking, whereas breast development may be present due to peripheral conversion of excessive testosterone (T) to estrogen. Radiological evaluation reveals the presence of testes in the abdominal cavity, while functioning Sertoli cells prevent the development of Müllerian duct derivatives (uterus, fallopian tubes). Occasionally, a rudimentary prostate gland may be detected. On the contrary, PAIS concludes a heterogeneous group of phenotypes ranging from ambiguous genitalia to mild hypospadias that may be caused by an identifiable AR gene mutation or by an error in AR signaling. Interestingly, identical AR mutations may display a wide spectrum of phenotypes [1]. Finally, MAIS may present as isolated impairment of spermatogenesis and infertility [2].

The diagnosis of AIS is based on detailed clinical, laboratory, and radiological evaluation [6]. It is supported by a 46, XY karyotype and by an hormonal profile of elevated T concentrations, accompanied by elevated Luteinizing Hormone (LH) due to impaired feedback of the hypothalamic-pituitary axis. In inconclusive cases, especially in prepubertal children, human Chorionic Gonadotropin (hCG) test can be applied in order to assess androgen production and exclude androgen biosynthetic defects [1]. The diagnosis is confirmed by the documentation of the relevant AR mutation in more than 95% of subjects with CAIS [3, 7]. However, in milder forms of the syndrome this is not the case, as mutation detection frequency varies from 28 to 73%. The underlying cause in cases with negative mutation analysis is speculated to be defects in postreceptor signaling [1, 3, 8]. Last but not least, the psychological aspect of the patient with AIS remains a very sensitive issue, given the lack of widely accepted approaching methods. Unlike previous beliefs, the current trend is that all medical information should be shared with the patient in an age-appropriate way [9]. Despite this “full sharing” approach, there are still challenges that have to be overcome, since the majority of the patients are not aware of their condition. These challenges include the development of effective communication skills by the healthcare team, the successful establishment of a therapeutic relationship, and the final delivery of a patient-centered care, focused on the unique circumstances of each patient. The gender assignment process and the consequent interventions must be accompanied by psychological support of the patient and its family members, as the psychosocial distress may be severe, especially in the case of PAIS. The aim of this double case report is to outline the difficulties that may rise during diagnostic approach of AIS, extending it even to more challenging aspects, including psychological stress that may result from gender assignment process, therapeutic interventions such as genital surgery (if any), and establishment of a solid and trustful communication between the health professionals and the young patient and its family.

## 2. Case  1

A girl of 11 years and 3 months was referred to our unit due to enlargement of the clitoris associated with obstructive symptoms at micturition. According to her prenatal history, amniocentesis was conducted due to advanced maternal age, revealing a 46, XY karyotype. Pregnancy was otherwise uncomplicated. Cesarean section was performed at the gestational age of 40 weeks and 3 days due to cephalopelvic disproportion and failure to progress. The newborn was a healthy full-term baby, without electrolyte imbalance, presenting with an inadvertent female phenotype. In order to assess the discordance between chromosomal and phenotypical gender, the SRY gene was examined and found to be present, while imaging of the brain, hypothalamus, and pituitary gland was normal. At that time-point, the family decided to take no further action.

Anthropometric characteristics at presentation were in the normal range for girls at the patient's age, while physical examination revealed excessive clitoridomegaly resembling a phallus of 6 cm and a shallow vaginal orifice. No testes were palpable. Pubertal maturation, pubic hair, breast, and axillary hair were of Tanner stage III, I, and II, respectively. Imaging of the lower abdomen with ultrasound and MRI revealed bilateral testicular tissue at the intraperitoneal space near the inner inguinal ring, hypoplastic penile cavernous bodies within a sizable clitoris, and the presence of a rudimentary prostate along with seminal vesicles. Basal hormones assessment showed elevated T (84.0 ng/dl) in the male reference range for the given pubertal stage, accompanied by mildly elevated LH (2.11 mU/mL) and follicular stimulating hormone (FSH) concentrations (18.56 mU/mL). Estradiol (E_2_) on the other hand was inappropriately low (14.45 pg/mL). An hCG stimulation test was performed in order to assess synthesis, conversion, and action of T and exclude other causes of 46, XY DSD, using the algorithm proposed elsewhere [7, 10]. The ratio of T to Δ_4_-androstenedione (Δ_4_A) was >0.8, excluding 17*β*-hydroxysteroid dehydrogenase-3 (17*β*-HSD-3) deficiency, while the ratio of T to dihydrotestosterone (DHT) was <20, excluding 5*α*-reductase deficiency. The high elevation of T concentration (ΔΤ) after the hCG stimulation (>100 ng/dl) indicated the presence of testicular tissue, excluded gonadal dysgenesis, and supported the diagnosis of AIS (Table 2). Imaging control revealed the presence of bilateral testicular tissue. The combination of almost female phenotype with minimal virilization and proper male gonadal function was supportive for the diagnosis of severe PAIS.

Reasonably, the assignment of female gender was recommended, since the patient had already been raised as a girl and the relevant interventions would be minimal. In addition, the testes that were found during imaging studies had to be removed, as they were atrophic and of increased risk for malignant change. Interestingly, the parents suggested that the child itself should be fully informed and participate in the final decision, and therefore, from the very first beginning, the patient was involved in all discussions made with the attending physicians. It is noteworthy that, being aware of the condition, she stated: “It does not matter if I am female or male, most of all I am a human being and thus gender assignment will not play any significant role in my future life.” Eventually, female gender was preferred by both the patient and the parents, and gonadectomy as well as cosmetic surgery of the external genitalia were successfully performed.

## 3. Case  2

A 3-year-old boy referred to our unit for further evaluation of micropenis, penoscrotal hypospadias, and a history of operated unilateral cryptorchidism. Regarding his perinatal history, progesterone was administered to his mother since 12 weeks of gestation due to placental detachment. Subsequently, intrauterine growth restriction (IUGR) was noticed and amniocentesis was performed, which demonstrated a normal 46, XY karyotype. Due to the persistence of IUGR and fetal circulation redistribution, cesarean section was performed at the gestational age of 33 weeks and 2 days (birth weight: 1130 g, birth length: 38 cm, head circumference: 28 cm) and the newborn was hospitalized in the Neonatal Intensive Care Unit for one month. Apart from the deformities of the external genitalia and right-sided cryptorchidism, the infant was normal at the time of discharge. He was referred for right orchidopexy, which was performed at the age of nine months.

At the time of presentation, somatometric characteristics were within normal range (height: 91 cm, weight: 12.5 kg, 10th to 25th percentile). Regarding external genitalia, microphallus was observed (length 2 cm) with penoscrotal hypospadias; the right testis was palpable in the scrotum, whereas the left one could not be detected. The rest of the physical examination was that of a prepubertal boy. Abdominal ultrasound revealed the left testis at the inner ring of the inguinal canal, while MRI of the hypothalamus-pituitary gland did not reveal any pathology of the sellar region. Basal concentrations of T and gonadotropins were in the prepubertal range. During GnRH stimulation test, a normal prepubertal response was documented. In addition, an hCG stimulation test was performed, showing a T/Δ_4_ ratio >0.8, T/DHT ratio < 20, and a ΔΤ > 100 ng/dl, findings that were indicative of androgen insensitivity (Table 3). Taking under consideration the clinical phenotype of undervirilization, combined with appropriateness for a toddler testicular function, the diagnosis of PAIS grade 3 was set.

What is remarkable in this patient is that undermasculinization of their child was such a stressful situation for the parents, that they subconsciously transferred their anxiety to their toddler. That was particularly evident since, during the few days that he remained hospitalized, the child very often repeatedly said “I am a young man.” Taking into consideration this context, the assignment of male gender was recommended. He was prescribed to receive chorionic gonadotropin 1500 units per week intramuscularly and was referred to the surgeons for unilateral orchidopexy as well as surgical repair of hypospadias. However, the follow-up was discontinued early and no further communication with the family was possible.

## 4. Discussion

The medical approach to AIS should comprise proper and timely diagnosis, gender assignment, a combination of surgical and conservative interventions, and establishment of a strong-based relation with the patient and its family. It demands a long-term management strategy by a multidisciplinary team composed of experienced specialists: pediatric endocrinologist, pediatric surgeon or urologist, gynecologist, clinical geneticist, neonatologist, and/or pediatric psychologist/psychiatrist, according to the needs of each patient [11]. Nevertheless, healthcare professionals that deal with AIS have to overcome several difficulties in order to fulfill the general concepts of care. Proper diagnosis is a challenge, since the majority of patients (especially those with PAIS) do not present a particular phenotypic pattern. In addition, gender assignment is cumbersome and needs to be justified bearing in mind a variety of factors, such as genitalia appearance, surgical options, and views of the family related to cultural, social, and religious beliefs [11].

The cases presented in this study illustrate some of the challenges mentioned above, especially those depending on parental experiences [12]. In the first patient, though being a case of severe PAIS, where female sex assignment and corresponding surgical/medical interventions are strongly recommended, there was a notable delay in the management due to the parents' preference for no intervention at the time when the problem was first recognized. Despite being aware of the discordance between karyotype and phenotype, no medical assistance was sought until the onset of puberty and the development of secondary male characteristics. As a result, they had to choose to involve their child in the gender assignment process, a fact that might increase dramatically the young adolescent's anxiety. Regarding the second case, even though the genital deformities were minimal and congruent with the karyotype, the patient's family was overwhelmingly stressed and avoided regular follow-up of their offspring.

Concerns have been raised on the assimilation of the discordance among chromosomal, phenotypic, and gonadal sex and its complications [8]. Despite the fact that little is known about the criteria of gender assignment in infants with diversity of external masculinization, the options are usually straightforward. Individuals with CAIS are raised as females. They conceptualize their psychosexual development as a female's one and, up to now, there is no observed dissatisfaction with their assigned gender. This might be explained by the effect of androgen unresponsiveness of the brain, in addition to unambiguous female sex of rearing [13]. On the other hand, many patients with PAIS are raised as males, as in case 2; the female gender is preferred only in selected cases with severe PAIS, as in the hereby presented case 1. Being 11 years old, the patient declared no preference to either male or female gender assignment. This is an unusual statement, as these patients are known to have female gender identity. Given the fact that the patient had already been raised as a girl until the time of referral, it is profound that a female gender identity may have already been developed in a subconscious way. A possible explanation for the patient's detached gender assignment declaration may be the parents' approach (the child should be fully informed, involved in all discussions made with the attending physicians, and participating in the final decision). Sex of rearing does not entirely depend on the degree of external masculinization, especially in cases of severe PAIS where there is no precise relationship between genotype and phenotype implying that other factors may influence the decision of the assigned gender. In some countries, the cultural factors define the sex of rearing, like in Asia where male gender is preferred even if the patient is severely undermasculinized. There is a clear need for more research on documenting phenotype, surgical procedures, and outcome criteria that will enhance gender assignment [1]. Unlike CAIS, psychological distress is more often seen in PAIS patients, irrespective of the choice of gender assignment. Almost 25% of individuals affected by PAIS suffer from identity crisis or dissatisfaction with the decided sex and, sometimes, a gender reassignment may be needed [14, 15]. Psychological support is mandatory. The decision for the assigned gender is made by the medical team and the family, depending on the aforementioned factors. There is also a controversy about the optimal time of gender assignment. Many specialists recommend the assignment to be decided the sooner possible, while others suggest watchful waiting until the age of 3 years, when it is believed that the gender identity begins to develop [3, 11].

Patients who are raised as females will need genitoplasty and gonadectomy (orchiectomy). Orchiectomy is essential, as the ectopically located testes carry a substantial risk of malignancy. This risk is higher in PAIS than in CAIS, with an incidence of 15% and even higher if the testes are located intra-abdominally [3, 7, 8, 11, 14]. Orchiectomy should take place before the onset of puberty, as virilization may take place, as in case 1, and complicate the management. Less severe cases of PAIS will have to undergo hypospadias repair and orchidopexy. The ideal timing is a highly debated topic, with the majority of the experts suggesting that between 6 and 12 months of age [16, 17]. Puberty may be medically induced at the desired timing towards the gender that has been assigned.

Another issue to be addressed is whether it is, from an ethical and psychological point of view, better or not to reveal the underlying condition to the patient himself. In the case of CAIS or severe PAIS, the patient who has been raised as a female has to cope with the fact that she is genetically male and that she will be infertile. In the past, the standard physicians' approach was not to reveal the whole situation either to the patient or to his relatives. Nowadays, according to relevant interview studies [7, 8, 11, 13, 14], the full or partial disclosure constitutes the first-line choice, abandoning the paternalistic approach of the past. Social and cultural factors may be important modifiers in this process, since different societies do not accept this patient-based approach. In any case, although the family has to be informed, the disclosure to the patient depends on both the child's maturity and the social characteristics of the family.

The EuroDSD consortium, the International Disorder of Sex Development (I-DSD) Registry, and the aforementioned AR gene mutation database are great international initiatives that have contributed a lot in this DSD entity, opening a new, promising path for a more holistic management [2, 7, 18, 19]. Nevertheless, longitudinal studies are urgently needed to clarify obscure points in the optimal management of AIS, such as quality of life, sex assignment, and long-term outcomes.

## 5. Conclusion

In conclusion, these case reports illustrate the obstacles that the healthcare team has to face and overcome when dealing with AIS patients. These two cases highlighted the wide phenotypical spectrum of the syndrome, presented the different procedure for a final assignment (according to age, phenotype, and related circumstances), and underlined the psychological aspect that is strongly affected. More studies need to be conducted in this field so that the provided care and support are more evidence-based.

## Figures and Tables

**Table 1:** Severity of androgen insensitivity.

Grade	Syndrome	Phenotype: genitalia	Phenotype: other
1	MAIS	Male	Infertility
2	PAIS	Male	Mild undermasculinization, hypospadias
3	PAIS	Male	Severe undermasculinization, cryptorchidism, bifid scrotum
4	PAIS	Ambiguous	Severe undermasculinization, phallus between penis and clitoris
5	PAIS	Female	Separate urethral and vaginal orifices, mild clitoridomegaly
6	PAIS	Female	Normal pubic/axillary hair
7	CAIS	Female	Scarce or absent pubic/axillary hair

CAIS: Complete Androgen Insensitivity Syndrome, PAIS: Partial Androgen Insensitivity Syndrome, MAIS: Mild Androgen Insensitivity Syndrome.

**Table 2:** hCG stimulation test in case 1.

Time (days)	Testosterone (ng/dl)	Δ_4_-Androstenedione (ng/ml)	Dihydrotestosterone (ng/dl)	Anti-Müllerian hormone (ng/ml)
0	84	2.0	18.6	0.899
1	526	1.8	50.3	—
2	645	2.5	58.9	—
3	532	2.2	60.8	—

hCG: human chorionic gonadotropin.

**Table 3:** hCG stimulation test in case 2.

Time (days)	Testosterone (ng/dl)	Δ_4_-Androstenedione (ng/ml)	Dihydrotestosterone (ng/dl)	Anti-Müllerian hormone (ng/ml)
0	<10	0.16	4.1	107.16
1	41	0.19	12	—
2	170	0.25	25	—
3	201	0.30	28	—

hCG: human chorionic gonadotropin.

## Additional Points

*Limitations.* Neither of the cases was genetically tested. Despite the fact that the yield is high in CAIS, it is low in PAIS.
